# Poumon du puisatier

**DOI:** 10.11604/pamj.2016.25.157.10824

**Published:** 2016-11-14

**Authors:** Amal Moustarhfir Elidrissi, Nahid Zaghba, Hanane Benjelloun, Najiba Yassine

**Affiliations:** 1 Service des Maladies Respiratoires, Centre Hospitalier Universitaire IBN Rochd, Casablanca, Morocco

**Keywords:** Poumon, puisatier, pneumoconiose, silicose, complication, Lung, well-digger, pneumoconiosis, silicosis, complication

## Abstract

Le puisatier a pour profession le creusement et l'entretien des puits pour fournir de l'eau. Il est au contact de divers minerais, particulièrement la silice, particule qui présente un risque certain de développement des maladies pulmonaires connues sous le nom de silicose. Le but de notre travail est de préciser le profil épidémiologique, clinique, radiologique et évolutif des patients puisatiers silicotiques. C'est une étude rétrospective concernant 54 cas de puisatiers ayant une silicose, colligés au service des maladies respiratoires du CHU Ibn Rochd de Casablanca, de Mars 1997 à Janvier 2016. Tous les malades étaient des puisatiers, de sexe masculin, avec une moyenne d'âge de 50 ans. Le tabagisme était retrouvé dans 36 cas et un antécédent de tuberculose était noté dans huit cas. La radiographie thoracique retrouvait des grandes opacités dans 39 cas, des petites opacités dans 15 cas, et un épaississement des septats dans 11 cas. Ce tableau de silicose s'était compliqué d'une surinfection bactérienne dans 37% des cas, d' un pneumothorax dans 4% des cas et d'une tuberculose dans 20% des cas. La prise en charge thérapeutique était celle des complications. La déclaration de la maladie professionnelle et de l'indemnisation était faite. L'évolution était bonne dans 12 cas, stationnaire dans 17 cas et mauvaise dans 16 cas. La silicose est une pneumoconiose fréquente chez les puisatiers. Elle retentit sur la fonction respiratoire. Nous soulignons l'association fréquente de tuberculose et nous insistons sur la prévention qui reste le meilleur traitement.

## Abstract (en)

The essential occupation of well-digger includes wells drilling and maintenance in order to provide water. He comes in contact with various minerals, especially silica, whose particles unquestionably are a contributing factor to pulmonary diseases known as silicosis. Our study aims to highlight the epidemiological, clinical, radiological and evolutionary features of well-diggers with silicosis. We conducted a retrospective study of 54 well-diggers with silicosis whose data were collected in the Department of Respiratory Diseases at the University Hospital Ibn Rushd of Casablanca from March 1997 to January 2016. All patients were male well-diggers with an average age of 50 years. Smoking was found in 36 cases and a personal history of tuberculosis was observed in eight cases. Chest x-ray showed large opacities in 39 cases, small opacities in 15 cases and septat thickening in 11 cases. Silicosis was complicated by bacterial infection in 37% of cases, by pneumothorax in 4% of cases and by tuberculosis in 20% of cases. Therapeutic approach was established based on these complications. A declaration of occupational disease and compensation was made. Outcome was good in 12 cases, stationary in 17 cases and poor in 16 cases. Silicosis is a common pneumoconiosis among well-diggers. It affects lung function. We here highlight its frequent association with tuberculosis and emphasize on prevention which is the best treatment.

## Introduction

La pneumoconiose est définie par des altérations causées par l'inhalation et la fixation dans le poumon de poussières minérales [1], susceptibles de provoquer une fibrose [2]. La silicose est la plus fréquente des pneumoconioses, due à l'inhalation de silice cristalline libre (SiO_2_) et fait partie des plus vieilles maladies professionnelles connues. Elle a été décrite pour la première fois par Visconti en 1870 [3]. La silicose se manifeste essentiellement chez les travailleurs exerçant une activité dans les exploitations minières souterraines. Elle se voit aussi dans les gravières et les carrières et dans l'industrie de transformation de la pierre. Elle peut s'observer chez les travailleurs dans l'industrie céramique, les fours industriels, les fonderies et dans l'industrie du verre et des matériaux de construction [4, 5]. Le puisatier est un artisan indispensable et incontournable pour les populations, présent dans toutes les sociétés. Si on le compare aux autres professions qui ont subi de nombreuses mutations à travers les siècles, le puisatier a gardé presque inchangé son art. Il est quotidiennement au contact de divers minerais, notamment la silice présente dans toutes les régions du continent africain [6], rendant ce métier très silicogène.

## Méthodes

Il s'agit d'une étude rétrospective, réalisée au service des maladies respiratoires du CHU Ibn Rochd de Casablanca entre Mars 1997 et Janvier 2016. Nous avons colligé 54 patients de sexe masculin avec une moyenne d'âge de 50 ans. Tous les patients avaient bénéficié d'un interrogatoire minutieux, d'un examen clinique complet, d'une imagerie thoracique, de recherche de BK dans les expectorations et d'une bronchoscopie souple. Les radiographies thoraciques standards ont été interprétées, selon la classification du Bureau international du travail (BIT) [7, 8]. Les petites opacités sont classées en fonction de leur diamètre en p (jusqu'à 1,5 mm), q (jusqu'à 3 mm) et r (jusqu'à 10 mm). Les grandes opacités en catégories : A (grand diamètre supérieur à 10 millimètres et inférieur ou égal à 50 millimètres), B (une ou plusieurs opacités de grand diamètre supérieur à 50 millimètres, la surface totale de ces opacités ne dépassant pas l'équivalent de la zone supérieure du champ pulmonaire droit) et C (une ou plusieurs opacités dont la surface totale excède l'équivalent de la zone supérieure du champ pulmonaire droit).

## Résultats

**Figure 1:**
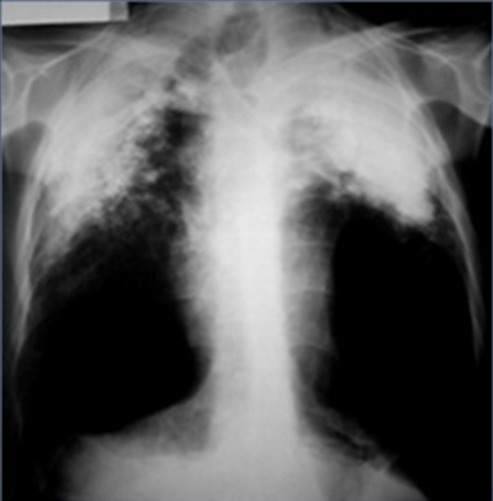
Radiographie thoracique montrant des opacités pseudo-tumorales hétérogènes bilatérales

**Figure 2:**
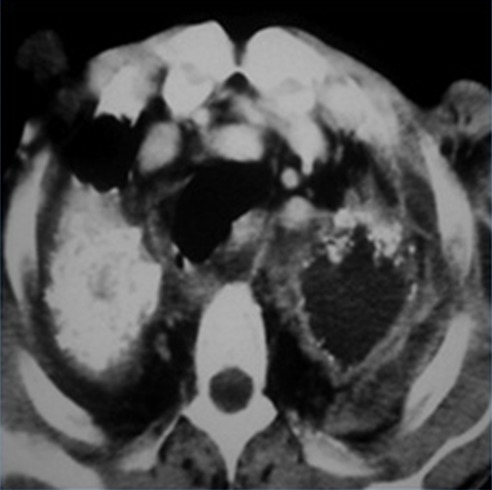
Scanner thoracique montrant des masses pseudo-tumorales bilatérales avec épaississement pleural

Tous les patients étaient des puisatiers, d'origine rurale, creusant manuellement des puits de 1,40 mètre de diamètre moyen et entre 20 et 38 mètres de profondeur. La durée moyenne de l'exposition était de 12,9ans. Seulement deux malades portaient des masques de protection lors de l'exposition. Ils étaient tous de sexe masculin avec une moyenne d'âge de 50 ans et des extrêmes allant de 34 à 82 ans. Le tabagisme actif a été retrouvé chez 36 patients et un antécédent de tuberculose a été noté chez huit malades. La symptomatologie clinique était faite d'une dyspnée dans 79% des cas, un syndrome bronchique purulent dans 67% des cas, une hémoptysie dans 38% des cas et un fléchissement de l'état général dans tous les cas. Selon les règles décrites par le BIT, la radiographie thoracique Figure 1) a retrouvé des petites opacités chez 15 patients (27,7%), des grandes opacités chez 39 patients (72%) et un épaississement pleural a été noté chez 11 patients (20,3%). Le type de chaque anomalie trouvée est détaillé dans le Tableau 1. Le scanner thoracique (Figure 2), réalisé chez tous les malades, a objectivé des opacités micronodulaires diffuses dans 29% des cas, un épaississement pleural dans 21% des cas, des adénopathies médiastinales calcifiées en coquille d'œuf dans 18% des cas, des bulles d'emphysème dans 66% des cas et un pneumothorax dans 4% des cas. La bronchoscopie avait objectivé un état inflammatoire dans tous les cas, avec des tâches d'anthracose dans 79% des cas et un épaississement des éperons dans deux cas. Le diagnostic de silicose était retenu devant l'ensemble d'arguments épidémiologiques, cliniques, radiologiques et l'exclusion d'autres diagnostics, notamment le cancer bronchique. Ce dernier reste le diagnostic à craindre vu le caractère cancérogène déjà connu de la silice, l'association fréquente au tabagisme et l'aspect pseudotumoral trompeur sur l'imagerie thoracique. Le tableau de silicose s'était compliqué d'une surinfection bactérienne dans 37% des cas, d'un pneumothorax unilatéral dans 4% des cas et d'une tuberculose dans 20% des cas dont 4% des cas avaient une rechute tuberculeuse. Le diagnostic de tuberculose pulmonaire sur tableau de silicose, ou communément appelé la Silico-tuberculose, a été retenu sur l'isolement des BK dans les expectorations chez 11 patients: neuf cas par l'examen direct avec coloration de *Ziehl-Neelsen*, deux cas par la culture sur milieu de *Lowenstein-Jensen* dont un cas dans les expectorations et l'autre cas dans le liquide d'aspiration bronchique. L'intradermoréaction à la tuberculine a été positive chez huit malades (72%).

**Tableau 1:** Types de chaque lésion radiologique trouvée

Lésions parenchymateuses ou pleurales élémentaires	Type de lésions	Nombre de malades	Pourcentage (%)
Grandes opacités	Catégorie C	39	72
Petites opacités	p	4	7,4
	q	7	12,9
	r	4	7,4
Epaississement pleural	b	11	20,3

Catégorie C = surface additionnelle supérieure à la zone droite. p = diamètre inférieur ou égal à 1,5 mm ; q = diamètre supérieur à 1,5 mm ; r = diamètre supérieur à 3 mm, inférieur à 10 mm. Epaississements pleuraux : b = épaississement maximal supérieur à 5 mm et inférieur ou égal à 10 mm

Dans le cadre du bilan de retentissement, une spiromètrie a objectivé un trouble ventilatoire mixte dans 67% des cas et un trouble ventilatoire restrictif probable dans 24% des cas. La gazométrie réalisée chez 15 patients était en faveur d'une insuffisance respiratoire chronique avec une hypoxie (type I) chez trois malades et avec une hypercapnie (type II) chez un patient. L'échographie cardiaque réalisée chez 21 patients a montré les signes d'hypertension pulmonaire dans 17% des cas avec une pression artérielle pulmonaire systolique moyenne à 46 mmHg (extrêmes : 30 à 65 mmHg). La prise en charge thérapeutique reposait sur le traitement des complications, notamment les antibacillaires dans 20% des cas, selon le régime 2SRHZ/4RH (deux mois de streptomycine, rifampicine, isoniazide et pyrazinamide, suivis de quatre mois de rifampicine et d'isoniazide) dans 6% des cas, et selon le nouveau régime du programme de lutte anti-tuberculeuse au Maroc de 2011 : 2RHZE/4RH (deux mois de rifampicine, isoniazide, pyrazinamide et d'ethambutol, suivis de quatre mois de rifampicine et d'isoniazide) dans 10% des cas, et le traitement de rechute tuberculeuse à base de 3RHZE/5RHE était indiqué dans 4% des cas. L'oxygénothérapie au long court était préconisée chez trois malades et la ventilation non invasive chez un malade présentant une insuffisance respiratoire type II. L'évolution était bonne dans 39% des cas et stable dans 15% des cas. Nous avons noté une aggravation de la fonction respiratoire chez 29% des malades dont la moitié est décédé. La silicose est une maladie professionnelle à déclaration obligatoire. Seulement deux de nos malades travaillant pour le compte de sociétés libérales ont bénéficié d'indemnisation.

## Discussion

La silicose est la maladie professionnelle la plus fréquente des pneumoconioses [9]. Elle fait partie des plus vieilles pathologies professionnelles connues et tue des milliers de personnes chaque année partout dans le monde. Elle est due à l'inhalation du quartz cristallin et ses formes cristallines (cristobalite et tridymite) [10]. Le quartz cristallin encore appelé silice cristalline libre (SiO2) est considéré comme la forme cristalline la plus répandue du dioxyde de silicium. Plusieurs métiers exposent à l'inhalation des particules de silice libre et la survenue des pathologies pulmonaires. Pour cela, plusieurs enquêtes épidémiologiques ont été réalisées chez les mineurs de fond, les perceurs de tunnels, les ardoisiers, les ouvriers de la métallurgie, les polisseurs et les travailleurs de la porcelaine, des faïences, du verre et de la céramique [11, 12]. Cependant peu de travaux ont concerné les puisatiers [13], d'où l'intérêt de notre travail.

Les facteurs déterminants pour le développement d'une silicose sont la diffusion alvéolaire, la concentration de la poussière de quartz, la durée de l'exposition à la poussière ainsi que divers facteurs individuels comme les antécédents d'infections des voies respiratoires (surtout la bronchite chronique et la tuberculose) et l'altération de la clairance mucociliaire. Chez nos patients, les facteurs de risque retrouvés étaient l'intensité et la durée de l'exposition (la durée moyenne d'exposition était de 12,9 ans) et la susceptibilité individuelle prédominée par la broncho-pneumopathie chronique obstructive (BPCO) post-tabagique dans 66% des cas et les séquelles de tuberculose dans 15% des cas. Dans notre étude, tous les malades étaient de sexe masculin avec une moyenne d'âge de 50 ans et une durée d'exposition moyenne de 12 ,9ans. Ces résultats sont comparables à ceux de Toloba Y et al. [14] au Mali, qui ont réalisé une étude concernant 39 cas de poumon de puisatiers. Tous étaient de sexe masculin avec une moyenne d'âge de 42 ,9ans et une durée d'exposition moyenne de 13ans. Selon une autre étude faite par Laraqui CH et al. au Maroc [10], la durée d'exposition moyenne à la silice était de 14,5 ans. Le tabagisme a été retrouvé dans notre série dans 66% des cas et selon les données de la littérature, les silicotiques tabagiques ont un risque plus important de développer un cancer pulmonaire par rapport aux silicotiques non tabagiques[15]. Leung CC. et al [16], trouvaient une relation significative entre le tabagisme et la tuberculose chez les patients présentant une silicose. Selon cette étude portant sur 435 silicotiques, l'incidence annuelle de la tuberculose est respectivement de 1,841, 2,294 et 4,181 pour 100.000 individus pour les non tabagiques, les ex tabagiques et les tabagiques actuels. Cette étude a conclue que les silicotiques fumeurs ont un risque plus élevé de développer la tuberculose que les silicotiques non fumeurs. Dans notre étude, on avait noté une tuberculose pulmonaire dans 20% des cas dont 6% des cas était des fumeurs. Par ailleurs, la silice, même sans silicose, peut aussi prédisposer les individus à la tuberculose [17, 18]. Ceci peut être du à la modification de la réponse immunitaire des poumons par les particules de la silice, l'altération du métabolisme et de la fonction des macrophages pulmonaires [19]. Plusieurs études ont montré que les symptômes cliniques prédominants chez les silicotiques étaient la dyspnée, la toux, les expectorations et la douleur thoracique. Dans notre étude, nous avons trouvé une dyspnée dans 79% des cas et un syndrome bronchique purulent dans 67% des cas.

La radiographie du thorax est un examen essentiel pour le dépistage et le diagnostic de la silicose. Habituellement, les lésions radiologiques élémentaires observées sont des opacités micronodulaires ou nodulaires, généralement bilatérales et symétriques, prédominant aux tiers moyens et supérieurs des deux champs pulmonaires. Elles sont caractérisées par leur taille, leur profusion et leur extension. Elles peuvent évoluer vers une confluence et réaliser des opacités de grande taille, dites pseudo-tumorales. Selon le BIT, ces lésions sont classées en fonction de leur taille, en petites et grandes opacités. Dans notre étude, on a trouvé une prédominance des grandes opacités dans 72% des cas. Les petites opacités à type de miliaire ont posé un problème de diagnostic avec la tuberculose comme c'était le cas pour d'autres auteurs [20]. Holanda M. et al avaient étudié chez les puisatiers, la relation entre l'exposition à la poussière et les lésions radiologiques en multipliant le nombre de puits creusés par le nombre de jours nécessaires. Il a défini ainsi un indice d'exposition à la poussière [13]. Dans notre étude, on n'a pas pu préciser cet indice, car la plupart des puisatiers n'avaient pas précisé le nombre exact de puits creusés. Les patients silicotiques peuvent présenter plusieurs complications rapportées régulièrement dans la littérature [21–26], notamment la tuberculose pulmonaire, les infections pulmonaires à mycobactéries atypiques, fongiques ou bactériennes, le pneumothorax, le cancer du poumon, la BPCO, les maladies rénales chroniques et les maladies auto-immunes notamment la sclérodermie (syndrome d'Erasmus) et la polyarthrite rhumatoïde (syndrome de Caplan Collinet). Il n'existe pas de traitement curatif de la silicose. Les lésions pneumoconiotiques aussi bien que les lésions emphysémateuses qui les accompagnent, sont irréversibles et ne peuvent qu'évoluer vers l'aggravation, malgré le traitement des complications. Ng TP. et al [27], trouvent que les facteurs prédicteurs de la mortalité chez les silicotiques sont : l'âge précoce d'embauche, la gravité de la silicose et la silicotuberculose. Dans notre étude, on avait déploré neuf décès (17%), dont deux malades avaient une silico-tuberculose et trois malades avaient un âge précoce d'embauche inférieur à 20ans. Quatre vingt dix neuf pour cent de nos patients travaillaient dans le secteur informel. Ils ignoraient tout ce qui concerne l'hygiène, la santé et la sécurité au travail, et ne bénéficiaient d'aucune assurance maladie. Il faut pourtant souligner qu'au Maroc, la silicose chez les puisatiers est prise en charge comme maladie professionnelle mentionnée au tableau 1.1.12 au bulletin officiel de 2014 [28]. Des actions d'information, d'éducation et de communication en faveur des puisatiers, tenant compte de leur degré d'analphabétisme et de leur faible niveau d'instruction, sur les risques encourus et l'hygiène générale sont nécessaires pour la démarche préventive [29].

## Conclusion

La silicose est une pathologie professionnelle à déclaration obligatoire, parfaitement évitable, grâce à des mesures de prévention visant l'éloignement des travailleurs de la source d'exposition, et la surveillance régulière des travailleurs exposés à la silice pour dépister les premiers cas de silicose et ses complications notamment la tuberculose [30].

### Etat des connaissances actuelles sur le sujet

- Les puisatiers sont exposés essentiellement à la silice;
- L'exposition à la silice présente un risque de développer une silicose;
- La silicose retentit sur la fonction respiratoire.

### Contribution de notre étude à la connaissance

- Précise le profil épidémiologique, clinique, radiologique et évolutif des patients puisatiers silicotiques;
- Souligne l'association fréquente de tuberculose;
- Insiste sur la prévention.

## References

[1] Brichet A, Tillie-Leblond I, Wallaert B (2000). Silicose et pneumoconiose du mineur de charbon. EMC Pneumol..

[2] Dai J, Gilks B, Price K (1998). Mineral dusts directly induce epithelial and interstitial fibrogenic mediators and matrix components in the airway wall. Am J Respir Crit Care Med..

[3] Greenberg MI, Waksman J, Curtis J (2007). Silicosis: a review. Dis--Mon DM..

[4] Rice F (2000). Crystalline silica, quartz..

[5] Terra Filho M, Santos U de P (2006). [Silicosis]. J Bras Pneumol Publicaçao Of Soc Bras Pneumol E Tisilogia..

[6] Leclerc J-C (1945). Structure et relief de l’Afrique occidentale. Études Rhodan..

[7] Zawilla N, Taha F, Ibrahim Y (2014). Liver functions in silica-exposed workers in Egypt: possible role of matrix remodeling and immunological factors. Int J Occup Environ Health..

[8] Rosental P-A (2008). La silicose comme maladie professionnelle transnationale. Rev Fr Aff Soc..

[9] Jalloul AS, Banks DE (2007). The health effects of silica exposure. Environ Occup Med..

[10] Laraqui CH, Laraqui O, Yazidi AA (2004). Évaluation des risques respiratoires chez les puisatiers de la région d’Agadir, Maroc. Arch Mal Prof Environ..

[11] Publique DS, LEGEAS M, Rennes E Une étude exploratoire et participative des retentissements du complexe minier de Sadiola au Mali. http://www.bape.gouv.qc.ca/sections/mandats/mines_malartic/documents/dc4.pdf.

[12] Le Bâcle C, Bouchami R, Goulfier C (1995). Silicose: la situation en France dans les années 90. Doc Pour Médecin Trav..

[13] Holanda MA, Holanda MA, Martins MP (1995). Silicosis in Brazilian pit diggers: relationship between dust exposure and radiologic findings. Am J Ind Med..

[14] Toloba Y, Sissoko BF, Badoum G (2014). Well-digger’s lung in Mali during the decade of 2001-2010. Rev Pneumol Clin..

[15] Westerholm P, Ahlmark A, Maasing R (1986). Silicosis and risk of lung cancer or lung tuberculosis: a cohort study. Environ Res..

[16] Leung CC, Yew WW, Law WS (2007). Smoking and tuberculosis among silicotic patients. Eur Respir J..

[17] Cowie RL (1994). The epidemiology of tuberculosis in gold miners with silicosis. Am J Respir Crit Care Med..

[18] Hnizdo E, Murray J (1998). Risk of pulmonary tuberculosis relative to silicosis and exposure to silica dust in South African gold miners. Occup Environ Med..

[19] Girling D (1979). Hong-kong-chest-service-tuberculosis-research-centre-madras-british-medical-research-council-study of 3-month and 2-month regimens for smear-negative pulmonary tuberculosis-results up to 2 years. british journal of diseases of the chest, Bailliere tindall 24-28 oval rd, london, england nw1 7dx..

[20] Snider DE (1978). The relationship between tuberculosis and silicosis. Am Rev Respir Dis..

[21] Fan Y-G, Jiang Y, Chang R-S (2011). Prior lung disease and lung cancer risk in an occupational-based cohort in Yunnan, China. Lung Cancer Amst Neth..

[22] Fernández Álvarez R, Martínez González C, Quero Martínez A (2015). Guidelines for the diagnosis and monitoring of silicosis. Arch Bronconeumol..

[23] Calvert GM, Rice FL, Boiano JM (2003). Occupational silica exposure and risk of various diseases: an analysis using death certificates from 27 states of the United States. Occup Environ Med..

[24] Weitzenblum E (2004). L’exploration fonctionnelle respiratoire en pneumologie..

[25] Pairon JC, Matrat M, Brochard P (2003). Mineral analysis of biological samples and respiratory pathologies. Rev Mal Respir..

[26] Devulder B, Plouvier B, Martin J-C (1977). The association: scleroderma-silicosis or Erasmus’ syndrome (author’s transl). Nouv Presse Med..

[27] Ng TP, Chan SL, Lee J (1992). Predictors of mortality in silicosis. Respir Med..

[28] Bulletin officiel Bulletin officiel. 12 moharrem 1436 (6-11-2014) Cent-troisième année..

[29] Graham WG (1992). Silicosis. Clin Chest Med..

[30] Gerhardsson G (2002). The end of silicosis in Sweden-a triumph for occupational hygiene engineering. OSH Dev..

